# A Case Report of Carotid Cavernous Fistula: A Commonly Missed Diagnosis

**DOI:** 10.5070/M5.52242

**Published:** 2026-01-31

**Authors:** Rosalind Wu Ma, Dustin Harris

**Affiliations:** * University of Texas at Southwestern, Department of Emergency Medicine, Dallas, Tx

## Abstract

Orbital compartment syndrome (OCS) is an ophthalmologic emergency due to an acute rise in intra-orbital pressure and can result in permanent vision loss, oftentimes requiring emergent surgical decompression. Orbital compartment syndrome most commonly occurs due to recent trauma and is often easily diagnosed by history and physical exam. However, there are other causes of OCS where a more subacute/chronic rise in pressure can lead to an atypical presentation. This is a case report of a 48-year-old male who presented with left eye pain and swelling for the past 6 months. The patient had seen two ophthalmologists prior to his emergency department (ED) presentation who had prescribed him an antibiotic ointment, oral steroids, and steroid eye drops. The physical exam in the ED was concerning for increased intra-ocular pressure (IOP) and decreased vision. Ophthalmology was consulted and requested magnetic resonance angiography (MRA) to assess a vascular etiology given tortuous retinal vasculature. Emergent surgical decompression was deferred due to chronicity of symptoms, and patient was started on dorzolamide/timolol and brimonidine eye drops as well as intravenous (IV) acetazolamide with subsequent improvement in IOP. Imaging revealed a left carotid-cavernous sinus fistula (CCF), and the patient was admitted to neurosurgery. Patient successfully had an embolization with subsequent normalization of IOP and improvement of symptoms. This case is a good example of how non-traumatic causes of OCS can lead to misdiagnosis and how surgical decompression could potentially be deferred in subacute OCS.

**Topics:**

Ocular compartment syndrome, carotid cavernous fistula, eye pain, eye swelling, vision loss.

**Figure:**
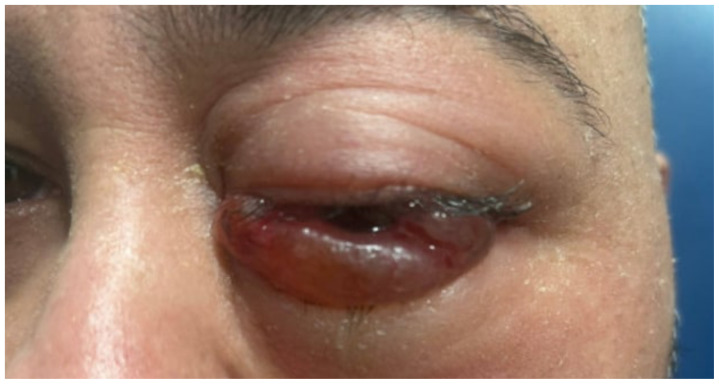


**Figure:**
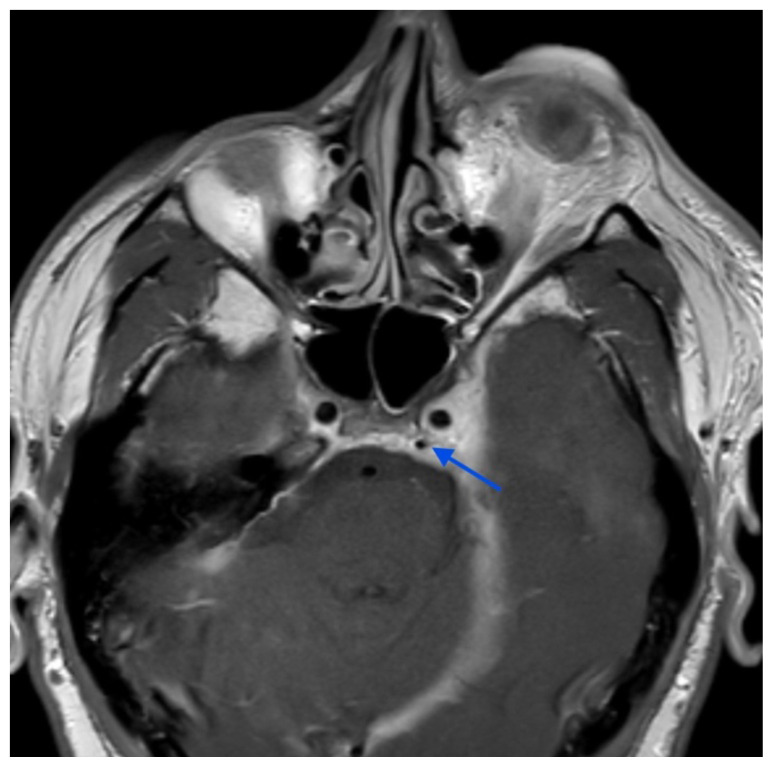


**Figure:**
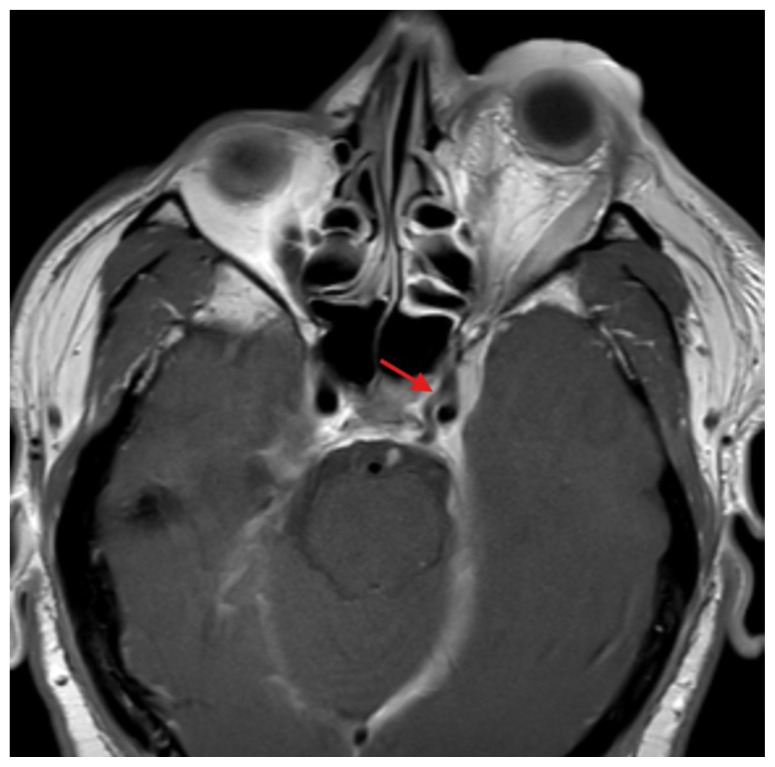


**Figure:**
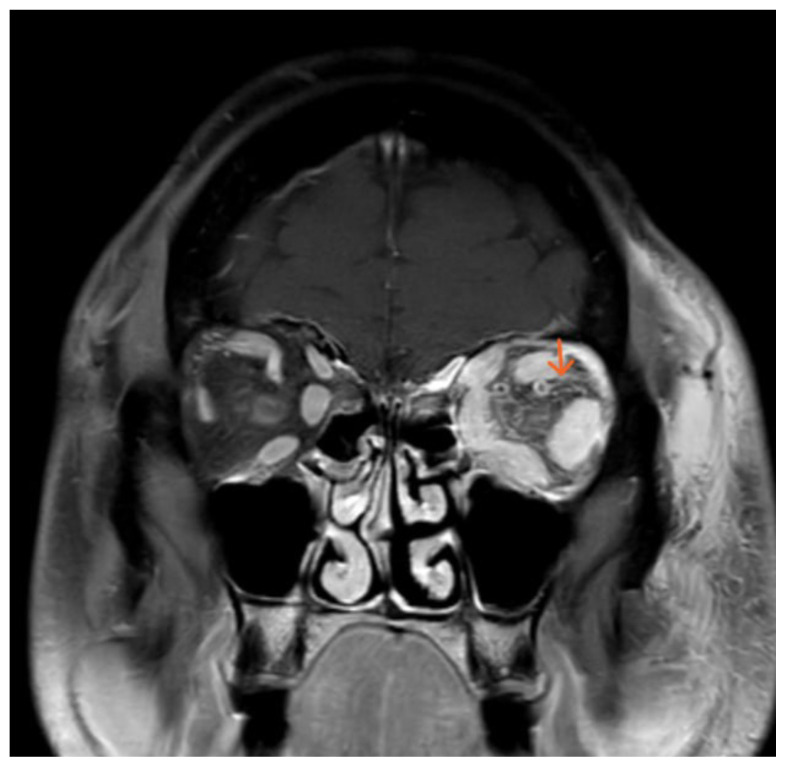


## Brief introduction

Of patients presenting to the ED, approximately 1.3% present with eye complaints.^1^,^2^ Eye pain is the most common eye chief complaint (approximately 79.8%), followed by swollen eye (7.8%).^2^ Orbital compartment syndrome is a rare ophthalmologic emergency that usually occurs due to an acute rise in intra-orbital pressure, resulting in damage to the optic disc and retina and oftentimes leading to permanent vision loss.^3^,^4^ Most common symptoms include reduced vision, increased prominence of the eye, and pain. It most commonly occurs after trauma and can frequently be diagnosed by history and physical exam. However, when there is a more subacute or slow rise in pressure, it is commonly misdiagnosed as infection or even as an allergic reaction, oftentimes resulting in initial mismanagement.

This is a case report focusing on CCF as a rare cause of OCS. The classic triad of CCF includes proptosis, ocular bruit, and chemosis.^5^ Endovascular intervention is the first-line treatment of CCF and has a cure rate of greater than 80%.^6^ Symptoms usually resolve within hours to days post-operatively. Although CCFs are usually not life-threatening, prompt treatment can prevent permanent injury to the involved eye.

## Presenting concerns and clinical findings

A 48-year-old male with a past medical history of hypertension, chronic kidney disease, tobacco use, and type 2 diabetes presented with worsening left eye proptosis and chemosis for the past six months. Patient reports he first noticed left eye redness and then developed fluctuating proptosis, swelling, and pain over the next few months. The patient denied history of trauma or head injury prior to the start of his symptoms. Prior to his ED visit, he had seen two different outpatient ophthalmologists who had prescribed him an antibiotic ointment, oral prednisone and ophthalmic steroids. The swelling worsened over the last week, prompting him to present to the ED. Patient denied significant vision changes. Written consent was obtained for publication of the images and case report.

## Significant findings

The initial physical exam performed by the ED provider revealed severe left eye chemosis, clear drainage, visual acuity of right eye 20/100 and left eye 20/400, and a left eye IOP of 52. Physical exam by the ophthalmologist also revealed that pupils were 3→2mm with brisk reflexes without relative afferent pupillary defect (RAPD) in the unaffected eye and 4 → 4mm with brisk reflexes with 2+ RAPD in the affected eye. Their assessment of IOP also revealed the affected eye had an elevated pressure of 47 obtained by handheld tonometer. There was a deficit of extraocular movement in all directions of gaze and limitation in all visual fields in the left eye. The slit exam of the left eye revealed proptosis, inability to close the eye completely (lagophthalmos), diffuse moderate chemosis (greatest inferiorly) and a deep quiet anterior chamber. The dilated fundus exam revealed vessels with increased tortuosity compared to the unaffected eye. The unaffected eye had an otherwise unremarkable ophthalmologic exam. The MRI showed that at the level of the eye, the left cavernous sinus is asymmetrically enlarged compared to the right (red arrow) with an enlarged left inferior petrosal sinus with internal flow void on the pre-contrast MRI images (blue arrow). The orange arrow notes a central filling defect of the left superior ophthalmic vein on the MRA.

## Patient course

Given the concerning ophthalmologic exam, ophthalmology was consulted. With their findings of tortuous retinal vessels as mentioned previously, intracranial vascular imaging was recommended. In the interim, ophthalmology deferred emergent surgical decompression given chronicity of symptoms and recommended brimonidine and dorzolamide/timolol eye drops as well as intravenous acetazolamide. Improvement of eye pressures from 47 to 31 was documented. Magnetic resonance angiography was obtained which revealed possible left carotid-cavernous sinus fistula and partial thrombosis of the left superior ophthalmic vein. Neurosurgery was consulted and the patient was admitted to their service. An urgent cerebral angiogram with embolization of the fistula was performed. A heparin drip was started post-operatively and IOP was improved to 14. There was also reported improvement in proptosis. The patient was ultimately discharged on daily aspirin 325 mg.

Follow-up visits in the ophthalmology clinic revealed a normal pressure of 7.4 in the left eye with full extraocular movement, no APD, and reported improvement of proptosis. The patient reported symptomatic improvement in pain and swelling as well.

## Discussion

This case report highlights how CCF can be misdiagnosed and also the ambiguity in literature on the recommended interim stabilization of IOP prior to definitive treatment of CCF. Carotid cavernous fistula is an abnormal connection between the cavernous sinus and the carotid arterial system.^7^ Common clinical presentations include ophthalmoplegia, a painful swollen eye, and changes in vision. The most common etiology of CCF is trauma, usually from a basal skull fracture resulting in a tear of the internal carotid artery within the cavernous sinus.^8^ However, spontaneous ruptures of existing aneurysms or atherosclerotic arteries is also possible and oftentimes present more insidiously.^9^ Diagnosis is usually confirmed with vascular imaging. While CTA is highly sensitive and specific for CCFs, MRA has still shown better overall diagnostic performance.^10^

Carotid cavernous fistula is oftentimes misdiagnosed due to its slow progressive and vague symptoms. In this case and in other documented case reports, patients oftentimes are treated as an allergic conjunctivitis or infection initially.^11^ Given the elevated IOP and physical exam findings, some providers may also consider glaucoma and retrobulbar hematoma as well. In emergency medicine, important eye examinations include visual acuity, intraocular pressure, extraocular motility, pupil exam, and visual fields. When the intraocular pressure is elevated and ophthalmoplegia is present, there should be a consideration of ophthalmologic consultation and/or advanced imaging to assess for retrobulbar pathology causing these exam findings.

While it is well established that the definitive treatment of CCF includes ligation of internal/external carotid arteries and fistula embolization, there is ambiguity on interim management of the patient’s pain and elevated IOP prior to surgical intervention.^7^ Anytime IOP is increased, orbital compartment syndrome and the potential risk of ischemia to the optic nerve should be a consideration. It is argued that OCS should be treated with surgical decompression even prior to imaging results.^4^ While most OCS cases are oftentimes acute, some cases have a delayed presentation and surgical decompression in these cases has also shown to improve visual acuity in the long term as well.^12^

In regards to CCF specifically, there have also been case reports where the patients were initially treated with surgical decompression and had improvement in pressures and symptoms.^13^ However, given that the elevated IOP in CCFs is primarily due to increased episcleral venous pressure from abnormal arteriovenous shunting resulting in impaired aqueous humor outflow, pressure reducing eye drops have been thought to be an effective therapy as well.^14^ Other cases, including this one, deferred decompression and opted for pressure reducing eye drops and IV acetazolamide, also resulting in a decrease of IOP of over 10 mmHg.^15^ While surgical decompression can immediately relieve pressure and reduce optic nerve ischemia, it does come with risks of globe rupture, bleeding, and infection.^16^ In cases where symptoms have occurred for a prolonged period and visual acuity is minimally affected, medical management may initially be preferred.

Overall, this case report highlights how CCF is a difficult diagnosis given its rarity and vague symptoms. It also highlights the ambiguity of IOP management in CCF prior to definitive care. We encourage all providers to perform a thorough ophthalmologic exam including IOP and extraocular movement and if abnormalities found, to consider ophthalmologic consultation and further imaging. While surgical decompression can be considered to relieve pressure in CCF, we argue that medical management can also be successful in decreasing eye pressure and improving symptoms.

## Supplementary Information












