# Supplementary figures and images for: A Case Report of Carotid Cavernous Fistula: A Commonly Missed Diagnosis
Source: J Educ Teach Emerg Med. 2026 Jan 31;11(1):V1–5. doi: 10.5070/M5.52242 (PMC12880886; doi:10.5070/M5.52242)

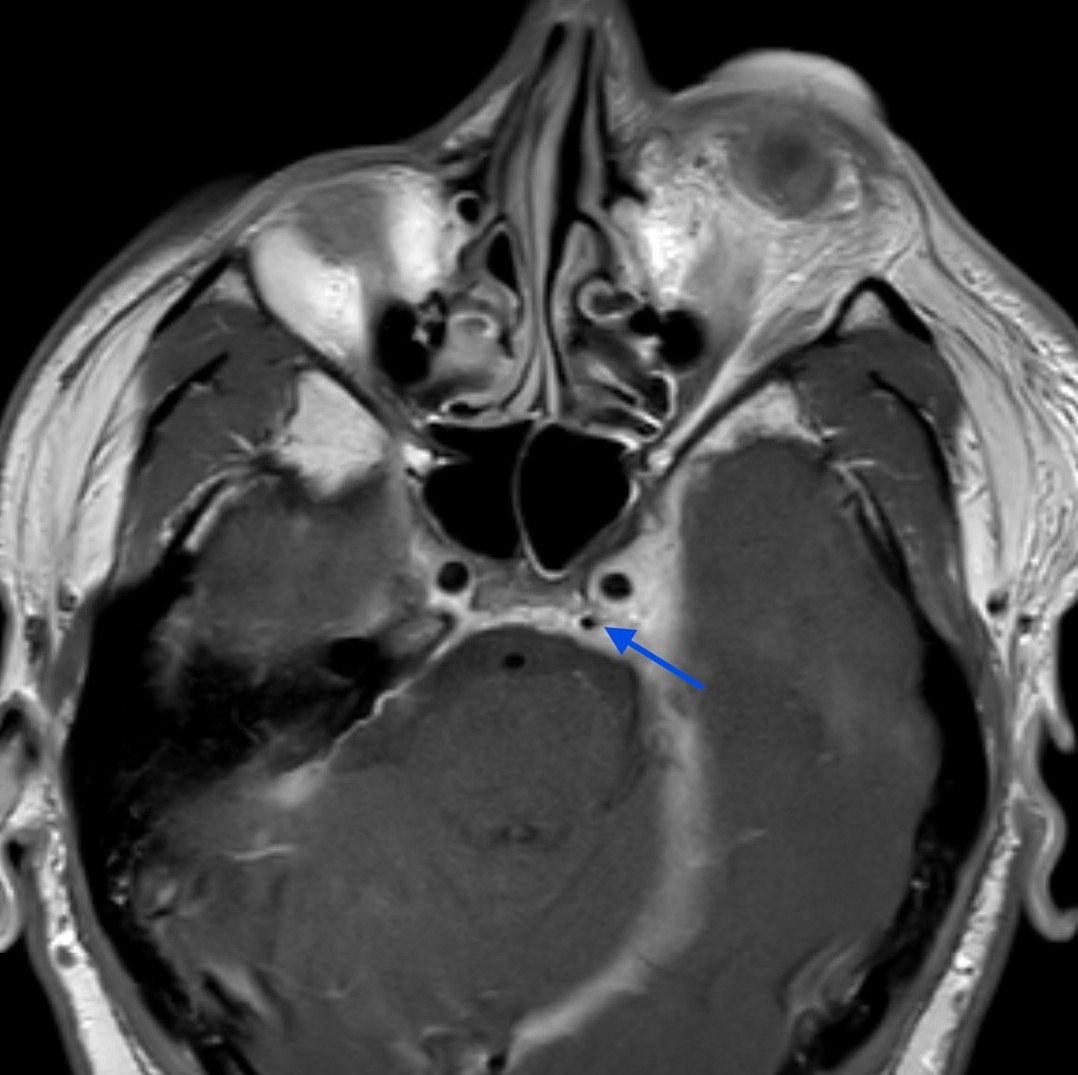

Supplement: Supplementary file 1 [file 11-1-V1-Supp1.jpg]

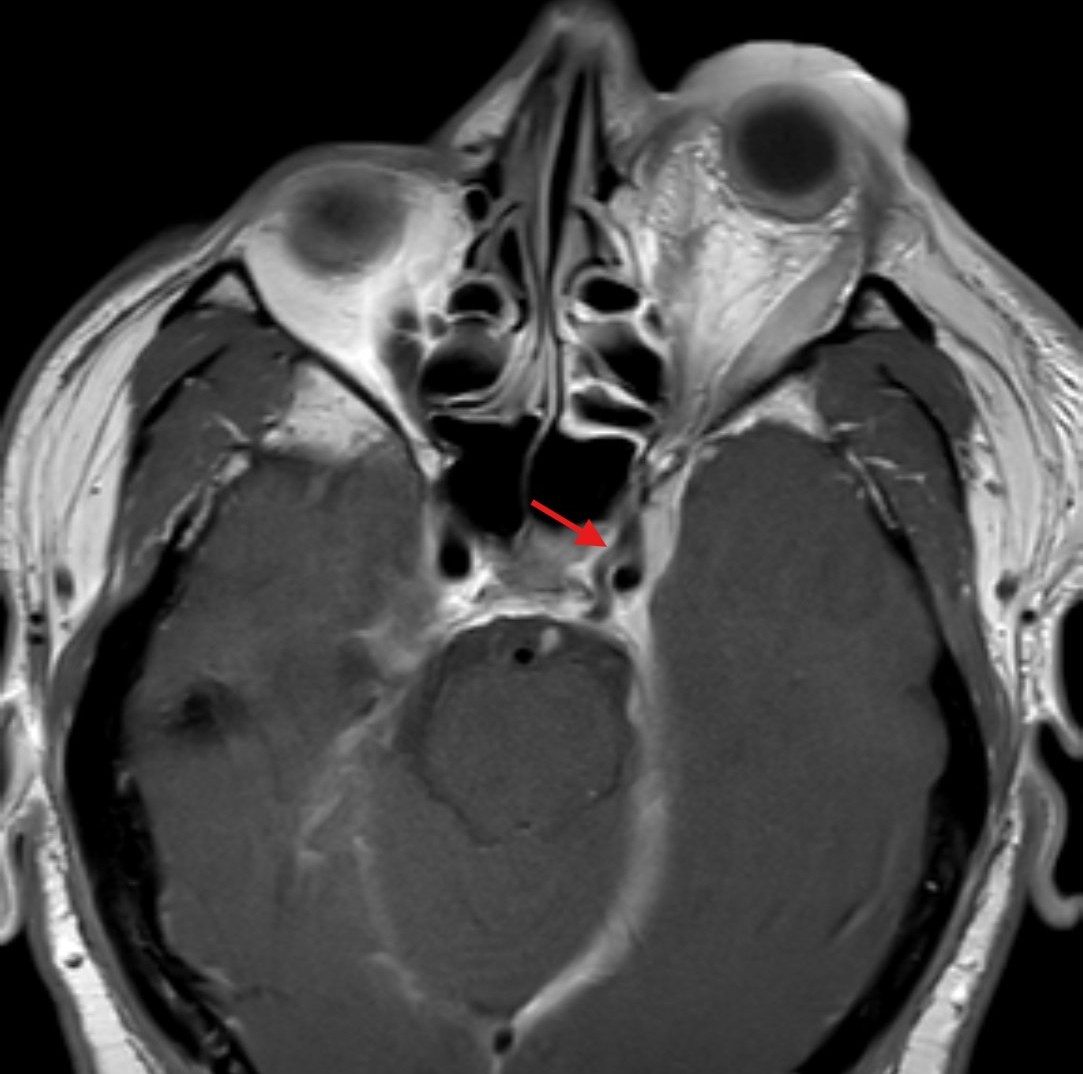

Supplement: Supplementary file 2 [file 11-1-V1-Supp2.jpg]

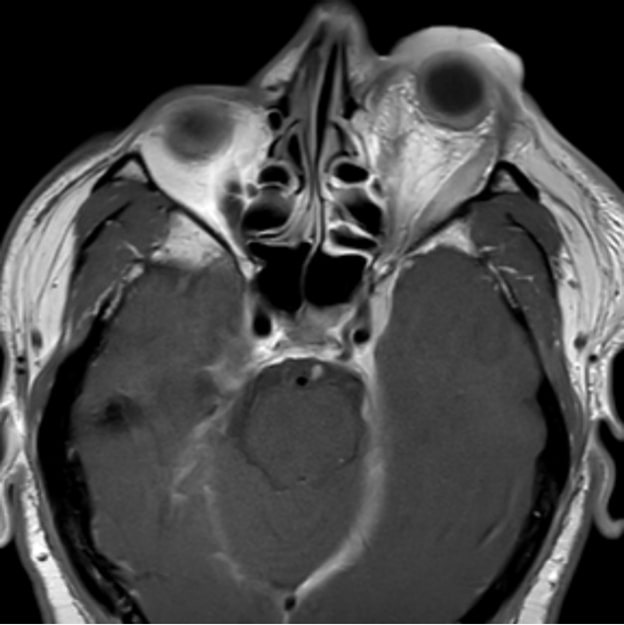

Supplement: Supplementary file 3 [file 11-1-V1-Supp3.jpg]

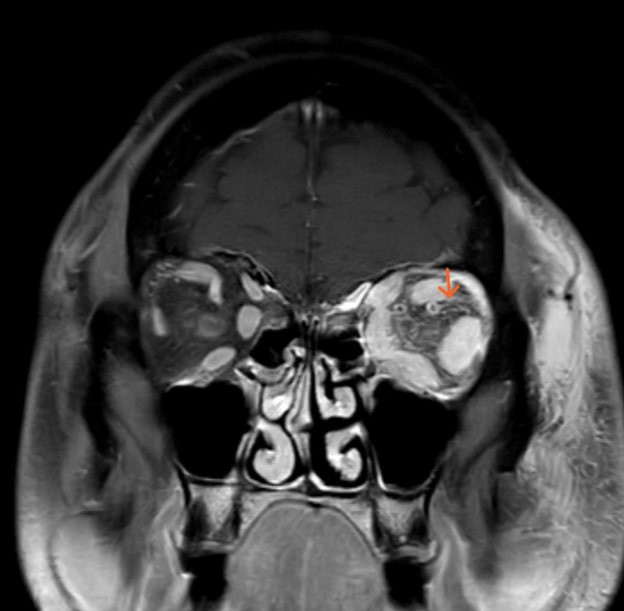

Supplement: Supplementary file 4 [file 11-1-V1-Supp4.jpeg]

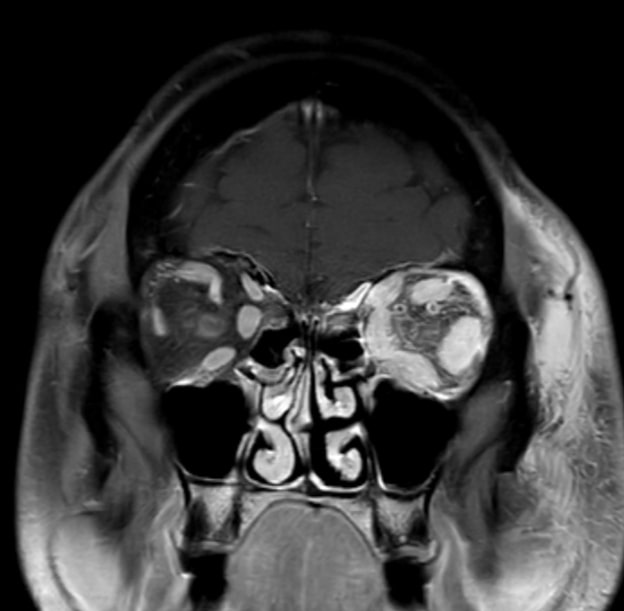

Supplement: Supplementary file 5 [file 11-1-V1-Supp5.jpg]

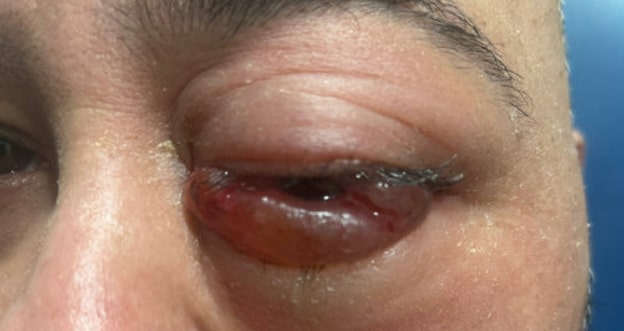

Supplement: Supplementary file 6 [file 11-1-V1-Supp6.jpg]
